# Assessment of sex-specific effects in a genome-wide association study of rheumatoid arthritis

**DOI:** 10.1186/1753-6561-3-s7-s90

**Published:** 2009-12-15

**Authors:** Joanna J Zhuang, Andrew P Morris

**Affiliations:** 1 Genetic and Genomic Epidemiology Unit, Wellcome Trust Centre for Human Genetics, University of Oxford, Roosevelt Drive, Oxford, OX3 7BN, UK

## Abstract

Rheumatoid arthritis (RA) is three times more common in females than in males, suggesting that sex may play a role in modifying genetic associations with disease. We have addressed this hypothesis by performing sex-differentiated and sex-interaction analyses of a genome-wide association study of RA in a North American population. Our results identify a number of novel associations that demonstrate strong evidence of association in both sexes combined, with no evidence of heterogeneity in risk between males and females. However, our analyses also highlight a number of associations with RA in males or females only. These signals may represent true sex-specific effects, or may reflect a lack of power to detect association in the smaller sample of males, and thus warrant further investigation.

## Background

The genetic contribution to rheumatoid arthritis (RA) susceptibility is undisputed, with replicated associations identified with haplotypes of the *HLA-DRB1 *locus and SNPs in *PTPN22 *[1,2]. The prevalence of the disease is approximately 0.8% in Caucasians, but is three times more common in females than in males [3], suggesting that sex may play an important role in modifying genetic associations with RA. The Wellcome Trust Case Control Consortium (WTCCC) performed genome-wide association (GWA) studies of 2,000 cases of each of seven common complex diseases, including RA, with a shared cohort of 3,000 controls [4]. In sex-differentiated analyses, RA was the only disease to demonstrate strong evidence of an effect in only one of the sexes, a female-specific association with inter-genic SNPs on chromosome 7q32.

Standard analyses of GWA data for both sexes combined may lack power to detect a sex-specific effect, particular if the direction of the association is different in males and females. Greater power may be achieved by analysis of males and females separately, although sample sizes will, of course, be smaller, or through sex-differentiated analyses in which both sexes are analyzed simultaneously, but allowing heterogeneity in male- and female-specific odds ratios [4].

Here, we investigate the hypothesis that sex may modify genetic associations with RA using data from a GWA study of 868 cases and 1,194 controls of Northern European ancestry in a North American population, genotyped using the Illumina HumanHap 550 k BeadChip. We perform sex-differentiated tests of association, and tests of sex interaction, and compare our results with those obtained from standard analyses for males and females combined. Our results demonstrate an association in the major histocompatibility complex (MHC), independent of the effect of *HLA-DRB1 *haplotypes, present in both males and females. We also highlight a number of apparent sex-specific effects, which may reflect false positives or a lack of power to detect association in males.

## Methods

Consider a sample of unrelated RA cases and unaffected controls typed for single-nucleotide polymorphisms (SNPs) in a GWA study. Let *G*_*i *_denote the genotype of the *i*^th ^individual at a SNP, coded as 0, 1, and 2 for the common homozygote, heterozygote, and rare homozygote, respectively. Assuming the effects on risk of the minor allele at the SNP act in a multiplicative fashion, we can model the log-odds of disease of the *i*^th ^individual in a logistic regression framework, given by

>(1)

In this expression, *s*_*i *_denotes the sex of the *i*^th ^individual (coded as 1 for males and 0 for females), and **x**_*i *_denotes a vector of additional covariate measurements, with corresponding regression coefficients *γ *and ***λ***, respectively, while the parameter *β *corresponds to the log-odds ratio of the minor allele at the SNP. We can perform a test of association of the SNP with RA, adjusting for the effects of sex and covariates, ***x***_*i*_, by comparing the fit of the model when *β *= 0 to that when *β *is unconstrained via analysis of deviance, having an approximate chi-squared distribution with one degree of freedom.

To allow for heterogeneity in allelic odds ratios between males and females, we can model the log-odds of disease of the *i*^th ^individual as

>(2)

where the parameter *θ *corresponds to the SNP-sex interaction effect. We can then construct a test of SNP-sex interaction, adjusting for the effects of covariates, ***x***_*i*_, by comparing the fit of the model when *θ *= 0 to that when *θ *is constrained via analysis of deviance, having an approximate chi-squared distribution with one degree of freedom. Furthermore, we can formulate a two-degree-of-freedom sex-differentiated test of association of the SNP with RA via analysis of deviance, by comparing the fit of the model when *β *= 0 and *θ *= 0 to that when both parameters are unconstrained [5]. When the genetic effect is different between males and females, this test provides greater power, in general, for detecting association than that based on *β *= 0 in Model (1).

## Results

Genotypes were reported for 531,689 autosomal SNPs to which stringent quality control (QC) filters were applied. A total of 35,111 SNPs were excluded from the analysis on the basis of low call rate (<97%) and extreme deviation from Hardy-Weinberg equilibrium (study-wide *p *< 5.7 × 10^-7 ^from exact test). To account for population structure and to minimize the effects of linkage disequilibrium, identity-by-state (IBS) metrics were calculated for each pair of individuals for every fifth SNP passing QC filters with study-wide MAF greater than 1%; the MHC was excluded to eliminate any bias due to the effect of *HLA-DRB1*. Application of multi-dimensional scaling techniques to the resulting matrix of pair-wise IBS statistics generated five axes of genetic variation associated with RA (*p *< 0.001) after adjustment for sex as a covariate.

For each SNP passing QC filters, we performed the following three tests.

• Test of association with RA assuming the same genetic effect for males and females, i.e., *β*≠0 in Model (1).

• Sex-differentiated test of association with RA allowing for heterogeneity of genetic effects between males and females, i.e., *β*≠0 and/or *θ*≠0 in Model (2).

• Test of interaction with sex, i.e., *θ*≠0 in Model (2).

In addition to sex, each test was adjusted for five axes of genetic variation and the number of shared epitope alleles to account for the effects of *HLA-DRB1 *haplotypes. SNPs with MAF less than 5% were excluded from the analysis because they have low power to detect modest genetic effects, particularly in sex-differentiated analyses.

Table 1 presents the lead SNPs in seven regions of strong association with RA (*p *< 10^-5^), assuming the same genetic effect for males and females. Also presented are allelic odds ratios (and 95% confidence intervals) for both sexes combined and for males and females separately, and *p*-values for the sex-differentiated test of association and test of sex interaction. The strongest signal of association is on chromosome 6, within the MHC, indicating an association independent of the effects of *HLA-DRB1 *haplotypes, with the same genetic effect on risk in both males and females. There is also strong association of SNPs in the previously identified *TRAF1-C5 *locus on chromosome 9, but with no apparent evidence of an effect in males. All other associations identified through this analysis are novel. For each of these SNPs, allelic odds ratios and confidence intervals for each sex suggested either no obvious heterogeneity in effects between the sexes (signals on chromosomes 16 and 20), or female-specific effects with no obvious association in males (signals on chromosomes 2, 17, and 18). However, there is no evidence of interaction with sex, indicating that any apparent variation in the strength of the association are more likely to reflect differences in the male and female sample sizes.

**Table 1:** Summary of single-SNP tests of association: SNPs demonstrating strong evidence of association (*p *< 1 × 10^-5^) of association with RA in the combined sex analysis

SNP	Chromosome	Location (Mb)	MAF	Combined sexes test		Allelic OR (95% CI)		Sex-differentiated test *p*-value	Sex interaction test *p*-value
							
				*p*-value	Allelic OR (95% CI)^a^	Females	Males		
rs6737562	2	180.05	0.065	7.11 × 10^-6^	2.30 (1.60-3.30)	2.44 (1.61-3.69)	1.77 (0.82-3.82)	2.72 × 10^-5^	4.56 × 10^-1^
rs17533090	6	32.70	0.189	1.58 × 10^-12^	2.38 (1.87-3.02)	2.36 (1.78-3.12)	2.42 (1.51-3.88)	3.85 × 10^-12^	7.76 × 10^-1^
rs2900180	9	120.79	0.340	3.23 × 10^-8^	1.68 (1.40-2.02)	1.87 (1.51-2.33)	1.25 (0.87-1.77)	2.22 × 10^-8^	4.86 × 10^-2^
rs4924	16	54.95	0.462	7.36 × 10^-7^	1.56 (1.31-1.86)	1.47 (1.20-1.80)	1.84 (1.30-2.61)	2.00 × 10^-6^	2.80 × 10^-1^
rs323413	17	69.24	0.410	6.53 × 10^-6^	1.49 (1.25-1.77)	1.59 (1.30-1.94)	1.25 (0.89-1.76)	1.42 × 10^-5^	1.98 × 10^-1^
rs9949777	18	2.10	0.306	7.57 × 10^-6^	1.52 (1.27-1.83)	1.62 (1.31-2.01)	1.31 (0.91-1.90)	1.94 × 10^-5^	2.61 × 10^-1^
rs1182531	20	57.83	0.190	2.76 × 10^-6^	1.76 (1.39-2.22)	1.76 (1.33-2.33)	1.78 (1.15-2.74)	1.23 × 10^-5^	8.71 × 10^-1^

^a^Assumes no heterogeneity of allelic odds ratios between males and females

Table 2 presents the lead SNPs in three regions of strong association with RA (*p *< 10^-5^), allowing for heterogeneity in genetic effects between males and females in the sex-differentiated test, not identified in the analysis of both sexes combined. The region identified on chromosome 11 showed some evidence of association in the combined sexes analysis, but demonstrated a stronger signal in the sex-differentiated test. There is an apparent effect in both sexes, with the same allele at risk in males and females. However, the risk appears to be greater in males than it is in females. The other two regions identified in this analysis demonstrated no evidence of association in the analysis of both sexes defined because the effects are in different directions in males and females, in other words the risk allele for one sex is protective in the other. Table 3 presents the lead SNPs five regions of stronger sex interaction (*p *< 10^-5^) not identified in the combined sexes or sex-differentiated association analyses. In each of these regions, the signal is stronger in males than females. In fact, in the region on chromosome 12 (~127 Mb), there is no association in females, suggesting a male-specific effect.

**Table 2:** Summary of single-SNP tests of association: SNPs demonstrating strong evidence of association (*p *< 1 × 10^-5^) of association with RA in the sex-differentiated analysis, not identified in the combined sex analysis

SNP	Chromosome	Location (Mb)	MAF	Combined sexes test		Allelic OR (95% CI)		Sex-differentiated test *p*-value	Sex interaction test *p*-value
							
				*p*-value	Allelic OR (95% CI)^a^	Females	Males		
rs7371994	3	54.57	0.289	5.30 × 10^-1^	1.06 (0.88-1.28)	0.76 (0.60-0.95)	2.36 (1.62-3.41)	5.09 × 10^-7^	1.68 × 10^-7^
rs12412942	10	2.35	0.403	1.91 × 10^-1^	1.13 (0.94-1.34)	1.47 (1.19-1.81)	0.53 (0.37-0.76)	1.49 × 10^-6^	7.08 × 10^-7^
rs17740690	11	122.90	0.065	3.43 × 10^-5^	2.19 (1.51-3.17)	1.65 (1.08-2.51)	5.33 (2.40-11.87)	4.63 × 10^-6^	1.13 × 10^-2^

^a^Assumes no heterogeneity of allelic odds ratios between males and females

**Table 3:** Summary of single-SNP tests of association: SNPs demonstrating strong evidence of sex interaction (*p *< 1 × 10^-5^), not identified in the combined sex or sex-differentiated analyses

SNP	Chromosome	Location (Mb)	MAF	Combined sexes test		Allelic OR (95% CI)		Sex-differentiated test *p*-value	Sex interaction test *p*-value
							
				*p*-value	Allelic OR (95% CI)	Females	Males		
rs3798014	5	9.49	0.398	5.45 × 10^-1^	1.05 (0.89-1.25)	1.38 (1.12-1.71)	0.56 (0.40-0.78)	1.50 × 10^-5^	3.98 × 10^-6^
rs7068778	10	95.01	0.167	6.17 × 10^-1^	1.06 (0.84-1.33)	0.77 (0.59-1.00)	2.80 (1.71-4.56)	2.07 × 10^-5^	5.24 × 10^-6^
rs33149	12	30.87	0.262	4.03 × 10^-1^	1.08 (0.90-1.31)	1.42 (1.13-1.78)	0.53 (0.36-0.77)	2.87 × 10^-5^	9.21 × 10^-6^
rs2699089	12	126.93	0.085	1.89 × 10^-1^	1.23 (0.90-1.66)	0.84 (0.59-1.19)	5.15(2.54-10.44)	1.20 × 10^-5^	6.03 × 10^-6^
rs677295	18	57.91	0.260	8.78 × 10^-1^	1.02 (0.83-1.24)	0.76 (0.60-0.96)	2.28 (1.51-3.43)	3.69 × 10^-5^	9.21 × 10^-6^

^a^Assumes no heterogeneity of allelic odds ratios between males and female

## Discussion

RA susceptibility has an undisputed genetic component, and also demonstrates a strong sex effect, with approximately three times higher prevalence in females than males. It is also one of the few diseases to have demonstrated a sex-specific association through GWA studies [4]. We have performed a range of tests of association and interaction to investigate the hypothesis that genetic risk for RA may vary with sex. Signals of association in the MHC, which are now well established for RA, demonstrate strong effects in both sexes. However, we have also identified a number of novel associations that demonstrate genetic effects in only one sex, or reciprocal effects on risk in males and females, although we do not replicate the female-specific association identified by the WTCCC [4]. The main disadvantage of these analyses is the small sample size, particularly in males. As a result, apparent female-specific effects may simply reflect a lack of power to detect association in males, particularly given the lack of evidence of sex interaction, which would be expected in the presence of heterogeneity of genetic risk between the sexes. Furthermore, the apparent male-specific associations occur at "low-frequency" SNPs (MAF<10%), and may reflect false positives due to genotyping errors. These considerations highlight the need for careful inspection of genotype calling quality, and then replication in follow-up samples from the same population.

Testing for the presence of sex-specific effects requires much larger sample sizes because males and females are analyzed separately, either directly or effectively, through interaction studies. To increase power, one solution is to combine results across GWA studies through meta-analyses of male- and female-specific effects. One obvious follow-up to the analysis presented here would be to combine the results of this study with that performed by the WTCCC [4]. Despite the fact that samples in the two studies have been genotyped using different technologies, results can be combined using imputation techniques [6]. Identification of sex-specific effects in this way may help to explain heterogeneity in results of association signals across GWA studies if ascertainment schemes vary with respect to the ratio of males and females sampled, and are thus extremely important in aiding our understanding of the biological processes underlying RA.

## List of abbreviations used

GWA: Genome-wide association; IBS: Identity-by-state; MHC: Major histocompatibility complex; QC: Quality control; RA: Rheumatoid arthritis; SNP: Single-nucleotide polymorphism; WTCCC: The Wellcome Trust Case Control Consortium.

## Competing interests

The authors declare that they have no competing interests.

## Authors' contributions

JJZ performed the statistical analysis. Both authors conceived of the study, drafted and approved the manuscript.
